# Whole Genome Sequencing Reveals Novel Non-Synonymous Mutation in Ectodysplasin A (*EDA*) Associated with Non-Syndromic X-Linked Dominant Congenital Tooth Agenesis

**DOI:** 10.1371/journal.pone.0106811

**Published:** 2014-09-09

**Authors:** Tanmoy Sarkar, Rajesh Bansal, Parimal Das

**Affiliations:** 1 Centre for Genetic Disorders, Faculty of Science, Banaras Hindu University, Varanasi, Uttar Pradesh, India; 2 Faculty of Dental Sciences, Institute of Medical Sciences, Banaras Hindu University, Varanasi, Uttar Pradesh, India; University of South Florida College of Medicine, United States of America

## Abstract

Congenital tooth agenesis in human is characterized by failure of tooth development during tooth organogenesis. 300 genes in mouse and 30 genes in human so far have been known to regulate tooth development. However, candidature of only 5 genes viz. *PAX9*, *MSX1*, *AXIN2*, *WNT10A* and *EDA* have been experimentally established for congenitally missing teeth like hypodontia and oligodontia. In this study an Indian family with multiple congenital tooth agenesis was identified. Pattern of inheritance was apparently autosomal dominant type with a rare possibility to be X-linked. Whole genome sequencing of two affected individuals was carried out which revealed 119 novel non-synonymous single nucleotide variations (SNVs) distributed among 117 genes. Out of these only one variation (**c.956G>T**) located at exon 9 of X-linked *EDA* gene was considered as pathogenic and validated among all the affected and unaffected family members and unrelated controls. This variation leads to p.Ser319Ile change in the TNF homology domain of EDA (transcript variant 1) protein. *In silico* analysis predicts that this Ser319 is well conserved across different vertebrate species and a part of putative receptor binding site. Structure based homology modeling predicts that this amino acid residue along with four other amino acid residues nearby, those when mutated known to cause selective tooth agenesis, form a cluster that may have functional significance. Taken together these results suggest that c.956G>T (p.Ser319Ile) mutation plausibly reduces the receptor binding activity of EDA leading to distinct tooth agenesis in this family.

## Introduction

Congenital tooth agenesis (CTA) is one of the most common cranio-facial disorders in human. Considering third molar (Wisdom teeth) agenesis, it affects up to 20% of the population. Among other teeth mandibular second premolar shows highest frequency of agenesis (2.9% to 3.2%) followed by maxillary lateral incisor (1.5% to 1.8%) and maxillary second premolar (1.4% to 1.6%).[1] Depending upon the severity, congenital tooth agenesis in human can be classified into three different forms: Hypodontia (Congenital absence of six or few teeth), oligodontia (Congenital absence more than six teeth), Anodontia (Congenital absence of all primary and/or permanent teeth).

Occurrence of hypodontia differs among population. A meta-analysis suggests that Saudi Arabian white people are less susceptible [2.5% (1.9% to 3.1%)] to tooth agenesis compared to North Americans [3.9% (3.1%–4.6%)], Europeans [5.5% (5.3%–5.6%)], Australians [6.3% (5.4%–7.2%)] and Chinese (Mongoloid) [6.9% (5.3%–.4%)]. It also shows clear gender bias; females are more susceptible for congenital tooth agenesis than males in all population except Saudi Arabia.[1] It has also been shown that most frequently missing tooth excluding 3^rd^ molars is mandibular second premolars (2.91%–3.22%) followed by maxillary lateral incisors (1.55%–1.78%) and maxillary second premolars (1.39%–1.66%) [1].

Congenital tooth agenesis in human may be syndromic as well as non-syndromic. In syndromic cases it is found to be associated with various forms of ectodermal displasia,[2], [3] oral-facial clefting like Pierre Robin syndome, [4] Van Der Woude syndrome [5] Hypodontia is also one of the cranio-facial features in several other syndromes such as Stickler’s syndrome, [6] Down’s syndrome [7], [8] and number of other syndromes [9].

Beside the syndromic cases congenital tooth agenesis is a well established birth defect that occurs as an isolated disorder. Non-syndromic tooth agenesis may be sporadic or familial. In familial cases it may show autosomal dominant, autosomal recessive or X-linked mode of inheritance. So far five major genes are found to be associated with CTA in human. These are Paired box 9 (*PAX9*) (MIM *167416),[10] Muscle segment homology homeobox 1 (*MSX1*) (MIM *142983),[11] Ectodisplasin A (*EDA*) (MIM *300451),[12] Axis inhibitor 2 (*AXIN2*) (MIM *604025),[13] Wingless type MMTV integration site family member 10A (*WNT10A*) (MIM *606268).[14], [15].

In the present study we have reported a novel non-synonymous mutation in *EDA* gene detected by whole genome sequencing and assessment of its pathogenic potential for determining the plausible cause for severe non-syndromic oligodontia (Tooth agenesis, selective, X-linked 1 MIM #313500) in an Indian family.

## Materials and Methods

### Family with congenital tooth agenesis

**Figure 1:**
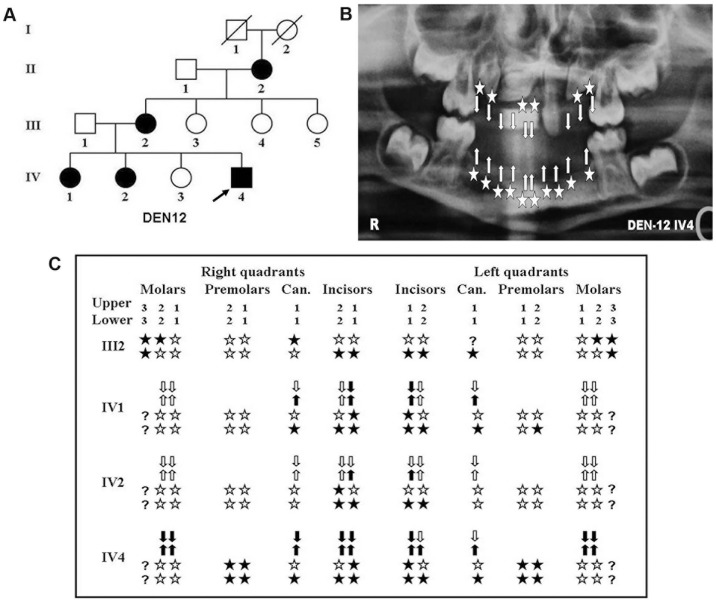
Pedigree structure and tooth agenesis pattern in affected individuals of DEN12 family. (A) Pedigree showing pattern of inheritance of congenital tooth agenesis in DEN12 family. (*squares* males, *circles* females, *blackened* affected, *clear* unaffected, *arrow* proband, *slash* deceased). Note that phenotype of deceased individuals is according to the memory of living relatives and phenotype of II2 of DEN12 is according to the information provided by the family members. (B) Representative orthopantomograph (OPG) of the proband showing the tooth agenesis pattern (*Star* missing permanent teeth, *arrow* missing deciduous teeth). (C) Deciduous and permanent dentition pattern of representative affected individuals. (*Arrow* position of deciduous teeth, *filled arrow* position of missing of deciduous teeth *star* position of permanent teeth, filled *star* missing permanent teeth). Note: status of the upper left canine (?) of DEN12 III2 is undetermined.

All the individuals investigated in this study belong to an Indian family (Figure 1A) showing multiple missing teeth. Following clinical diagnosis of the individuals as oligodontia patients at the faculty of Dental Sciences, Banaras Hindu University, an informed written consent was signed from patients and their unaffected family members for their participation in the present study. For minors (bellow 18 years of age) written consent was signed by their parent for their participation in this study. The study protocol and subject consent were approved by Institutional Review Board of Banaras Hindu University (No. Dean/2008-09/484). The severity of tooth agenesis was determined using the orthopentamogram (OPG). All available patients were critically screened for any other congenital disorders related with hair, skin, nails, tooth and other birth defect by interview. Blood samples were collected in heparinised syringe from four affected and two unaffected family members of DEN12 family.

### Whole Genome sequencing

Genomic DNA was extracted from whole blood using the protocol introduced by Grimberg *et al.* [16] with slight modifications and purified by standard phenol-chloroform-isoamyl alcohol method. Two affected members of DEN12 family were selected for performing whole genome sequencing. Genomic DNA sample was processed for library preparation using TruSeq DNA Sample Preparation Kit (Illumina) and sequencing was performed using Illumina HiSeq 2500 platform with an average of 10X depth; base calling was performed using CASAVA software v1.8.2 (Illumina) and variant calling was performed based on Human reference Sequence v19 (NCBI) database using Avadis NGS v.1.5.1 (Strand scientific intelligence, Inc).

### DNA Sequencing

To screen *PAX9* and *MSX1*; exon 2 to 4 along with exon-intron boundaries and a previously reported putative promoter region [chr14:37129645-37130161, refNC_000014.8, *H. sapiens* chr 14, GRCh37.p10 Primary Assembly] of *PAX9* and exon 1 through 2 along with exon-intron boundaries of *MSX1* were initially amplified from four affected and two unaffected individuals of DEN12 family using polymerase chain reaction (PCR). PCR was carried out using 50 ng genomic DNA in ABI Veriti 96 well thermal cycler (Applied Biosystems, USA) programmed with initial denaturation at 95°C for 5 min., followed by 30 cycles of 94°C for 1 min., 60°C to 62°C for 1 min., 72°C for 1 min. and one final hold at 72°C for 10 min. All the PCR products were purified by exonuclease I and recombinant Shrimp alkaline phosphatase (rSAP) (USB Affimetrix, USA). All the primers for amplifying *PAX9* putative promoter, *PAX9* and *MSX1* exon were designed previously by Mendoza-Fandino *et al.* (2010),[17] Das *et al*. (2003)[18] and Wang *et al.* (2011)[19] respectively. PCR products were labeled with ABI Big Dye Terminator V3.1 cycle sequencing kit followed by automated sequencing in ABI 3130 Genetic Analyzer according to manufacturer’s protocol. All the sequences were analyzed using Sequencing analysis software version 5.2 (Applied Biosystems, USA). To identify any sequence variation(s), all the sequences of putative promoter region, exon 2 to 4 of *PAX9* and exon 1 to 2 of *MSX1*, were compared with available National Center for Biotechnology Information (NCBI) GenBank database using NCBI- Basic Local Alignment Search (BLAST) tool.

For genotyping c.956G>T variation of *EDA* gene (NM_001399), primers h*EDA*ex8F1 (5′-GATTCTGTCAATTCACCACAG-3′) and h*EDA*ex8R1 (5′-ATCTTGACGGCGATCTT CTG-3′) were designed using Primer3web v4.0.0 software and used to amplify and sequence (Sanger dideoxy chain termination sequencing) an amplicon of 250 bp, harboring the variant 115 bp downstream from 5′ side of the amplicon, from all the affected and unaffected individuals of the family.[20], [21] The PCR and sequencing protocol is same as mentioned above.

### Genotyping of c.956G>T nucleotide variant

For cross validation of c.956G>T variant detected in *EDA,* PCR-Restriction Fragment Length Polymorphism (PCR-RFLP) and Single-Strand Conformational Polymorphism (SSCP) were designed using the same 250 bp amplicon. For SSCP amplicon from case and control samples were denatured at 95°C for 5 min in the presence of denaturing buffer (5% formamide; 0.01N NaOH; 20 mM di-Na-EDTA, pH8.0; 0.05% bromophenol blue and 0.05% xylene cyanol) followed by rapid chilling on ice. Electrophoresis was performed using 10% non-denaturing midi (14 cm×15 cm) polyacrylamide gel containing 10% glycerol and 1× TBE at 25°C for 13 hr under 250V. Gel was stained using standard silver staining protocol. For PCR-RFLP, 250 bp amplicons from patients and controls were digested using 3U of *Alu*I restriction endonuclease (New England BioLabs Inc.) at 37°C for 8 hr. Digested products were resolved using 4% agarose gel and stained with Ethidium Bromide.

### *In silico* analysis of EDA1 c.956G>T (p.Ser319Ile) and its effect on protein structure

In order to determine the inheritance pattern of X-linked *EDA* c.956G>T variant, sequences from all the affected and unaffected individuals through Sanger sequencing were aligned using NCBI-BLAST tool. PolyPhen-2 (Polymorphism Phenotype v2.2.2) tool was used to predict pathogenic potential of p.Ser319Ile mutation in EDA1 protein (NP_001390.1).[22] Multiple sequence alignment of the wild type and mutated EDA1 protein (UniProtKB/Swiss-Prot: Q92838.2) with Protein Data Bank (PDB) was carried out using blastp algorithm of NCBI BLAST tool. Subsequent conserved domain was mapped using NCBI Conserve Domain Database (CDD) [23], [24].

Further analysis for assessment of probable consequence/s of Ser319Ile mutation on EDA1 function, three dimensional model has been created using PDB atomic coordinate file (1RJ7) for EDA1 protein [25]. Four amino acids (Gln331, Met364, Val365 and Ser374) for which mutation has been known to cause non-syndromic tooth agenesis and Ser319 (present study) were used to create stick model; surface electrostatic potential for those residues were also calculated. All of this modeling was performed using Swiss-PdbViewer 4.1.0 tool [26].

## Results

### Clinical findings and mode of inheritance

In this family (DEN12), six years old boy (IV4), his two elder sisters (IV1 and IV2), who were under age of 12 years, and his mother (III2) showed non-syndromic incisors-canine tooth agenesis while his father (III1) and another elder sister (IV3) were unaffected. The pattern of inheritance was seems to be autosomal dominant with a rare possibility of being X linked dominant type (Figure 1A). All the affected children showed deciduous as well as permanent teeth agenesis. The 6 years old boy, who was the proband, was most severely affected than others. All mandibular deciduous and permanent teeth except first and second permanent molars were missing. Among maxillary deciduous dentition, all but one incisor teeth were missing. Among maxillary permanent teeth two incisors and all four premolars were missing. One maxillary permanent incisor was larger in size as appeared in OPG (Figure 1B). However, two of his elder sisters were less severely affected. In one of his sisters (IV1) all the deciduous maxillary and mandibular central incisors along with mandibular canines were missing. Regarding permanent teeth all her mandibular incisors, canines, one second premolar and two maxillary central incisors were missing. In his other sister (IV2) two mandibular deciduous central incisors, all four mandibular permanent incisors and one maxillary permanent lateral incisor were missing. However, the site for tooth agenesis in their affected mother (III2) confined to all mandibular incisors, one mandibular and one maxillary canine along with both the maxillary second molars and all third molars (Figure 1C).

**Figure 2:**
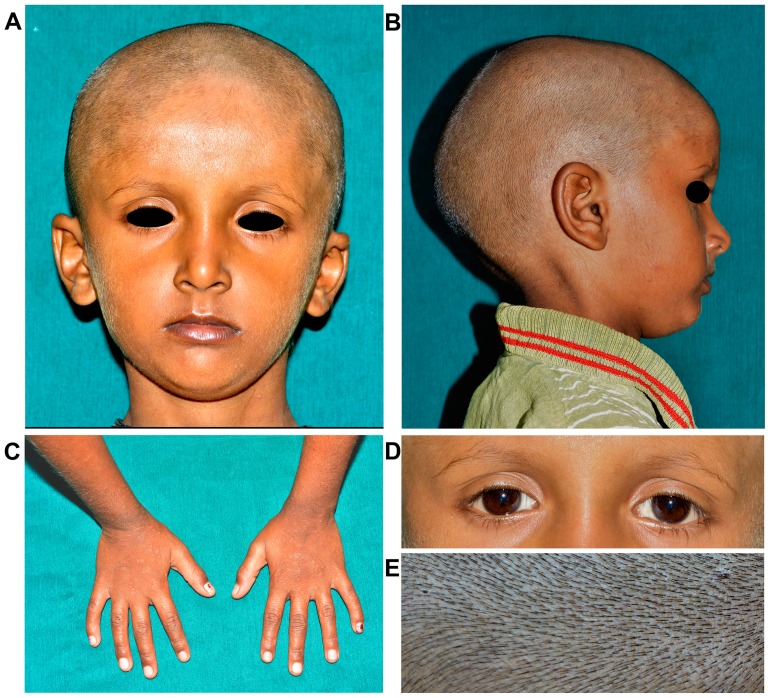
Facial and dermatological features of the proband (DEN12:IV4). (A–B) Frontal and lateral view of proban’s face. (C) Hands (D) Eyes and (E) Scalp hair.

Besides tooth agenesis scalp hair density, body hair, eyes, nail, skin and facial appearance of the proband and all the affected individuals were normal. There was no family history of thermal intolerance and problem in perspiration (Figure 2).

### Whole genome sequencing

**Figure 3:**
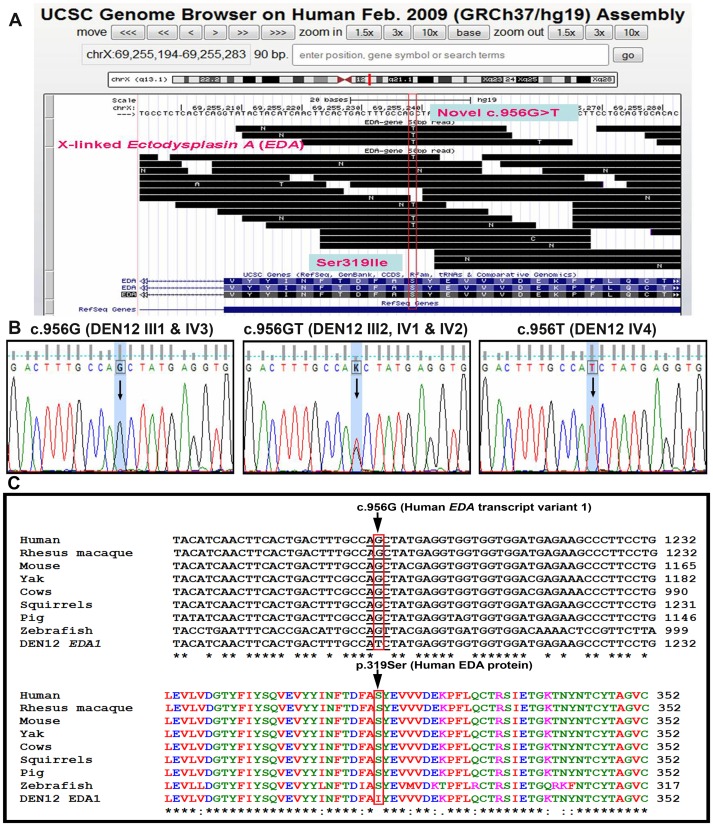
Novel mutation identification at evolutionarily conserved c.956/p.319 position of *EDA*. (A) Over view of 50 base pair reads, generated through whole genome sequencing, from patient samples flanking c.956 position located in *EDA* exon 9. Highlighted ‘T’ base indicating the mismatched base at c.956 position when aligned to human genome data base using UCSC Genome Browser. (B) Representative chromatogram of Sanger sequencing confirming nucleotide transversion form G to T at c.956 in exon 9 of *EDA1* gene identified through whole genome sequencing in affected members of DEN12 family. (C) Multiple sequence alignment of a portion of TNF-homology domain of human EDA1 orthologs and mutant EDA1 (DEN12- EDA1) reported in this study at mRNA lavel (upper panel) and protein lavel (lower panel) showing conserved c.956G and p.319Serine across different vertebrate species.

50 bp reads generated through whole genome sequencing was initially filtered through dbSNP (NCBI) database which remove all the annotated single nucleotide polymorphisms, intronic, intergenic and synonymous changes were also filtered out. In all 119 novel non-synomous variants, distributed in 117 different genes, were detected which were unique to this two affected individuals [Supplementary data file (Table S1)]. Further screening for all the known candidate genes for congenital non-syndromic tooth agenesis viz. *PAX9*, *MSX1*, *AXIN2*, *WNT10A* and *EDA*, only one novel non-synonymous variant resulting in G to T transversion was identified at c.956 position (NM_001399) of *EDA* gene exon 9 (Figure 3A) leading to p.Ser319Ile (UniProtKB/Swiss-Prot: Q92838.2) change.

### Exclusion of *PAX9* and *MSX1* genes and analysis of c.956G>T mutation status in all the cases and controls

Through NCBI-BLAST and UCSC-BLAT search, no pathogenic mutation had been identified in all the coding exons and exon-intron boundaries of *PAX9* and *MSX1* genes except a few reported polymorphisms (data not shown).

**Figure 4:**
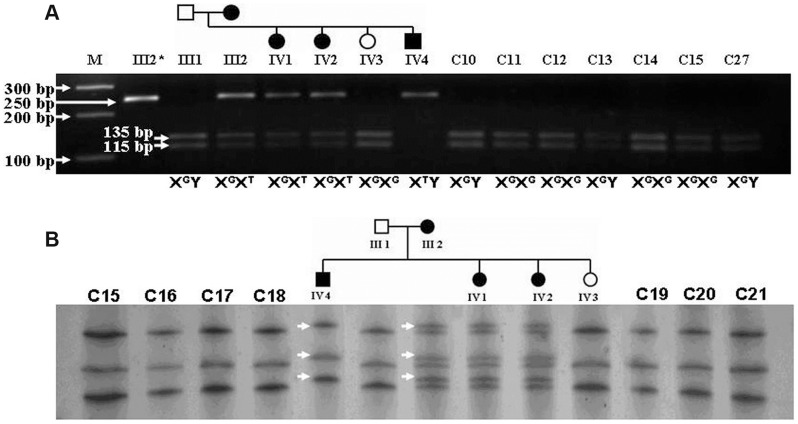
SSCP and PCR-RFLP analysis of c.965 G>T change in *EDA*. (A) Representative SSCP gel picture showing abnormal band (*white arrow*) co-segregated with disease phenotype which are absent in normal individuals. (B) PCR-RFLP pattern after resolving Alu I digested 250 bp PCR product which generate 135 bp and a 115 bp fragment. * indicate undigested PCR product.

The c.956G>T mutation (ClinVar ID SCV000148371 and dbSNP ID rs483352804) in *EDA*, detected through whole genome sequencing, was cross validated using Sanger sequencing in all the affected and unaffected individuals of the family. This change was present in heterozygous (X^G^X**^T^**) condition in all three affected females (III2, IV1 and IV2) and in the affected male (IV4) as hemizygous (X**^T^**Y) condition (Figure 3B). None of the unaffected individuals (III1 and IV3) had the mutant T allele. Further RFLP and/or SSCP were also used to cross validate the findings. 104 normal unrelated controls with same ethnic background which include 18 females and 86 males with 25 to 30 years age group with all the affected and unaffected individuals were screened using allele specific PCR-RFLP. A 250 bp amplified product of *EDA* exon 9 encompassing this said DNA variation get digested into 115 bp and 135 bp products with *Alu I* in presence of wild type allele (G) while presence of mutant allele (T) destroys the *Alu I* restriction site resulting only 250 bp product. (Figure 4A). In SSCP distinct polymorphic bands were observed associated with disease phenotype (Figure 4B). These two assays showed that this nucleotide variation was segregated with disease phenotype only and was not present in any of the unaffected family members and 104 unrelated normal control individuals suggesting this nucleotide variation (c.956G>T) as a novel and potentially pathogenic mutation.

### *In silico* functional characterization of novel *EDA1* mutation

*In silico* analysis using UCSC genome browser and NCBI data base showed that *EDA* c.956G>T change caused a substitution from uncharged polar amino acid Serine to hydrophobic non-polar Isoleucine at 319 position of EDA transcript variant 1 (EDA1). Multiple sequence alignment of human *EDA1* mRNA and amino acid sequences with its orthologs using ClustalW2 software, showed that the c.956G and Ser319 residue is evolutionary conserved across different vertebrate species (Figure. 3C). PolyPhen-2 tool denotes EDA1 Ser319Ile mutation as potentially damaging. Multiple sequence alignment with Protein Data Bank proteins (PDB) database showed that Ser319 is one among the five residues maintaining the conserved feature of receptor binding site of TNF homology domain of EDA. NCBI-CDD analysis showed that due to the mutation of p.319 Ser to Ile number of residues maintaining the conserved feature domain for receptor binding was reduced down from five to four (Data not shown).

**Figure 5:**
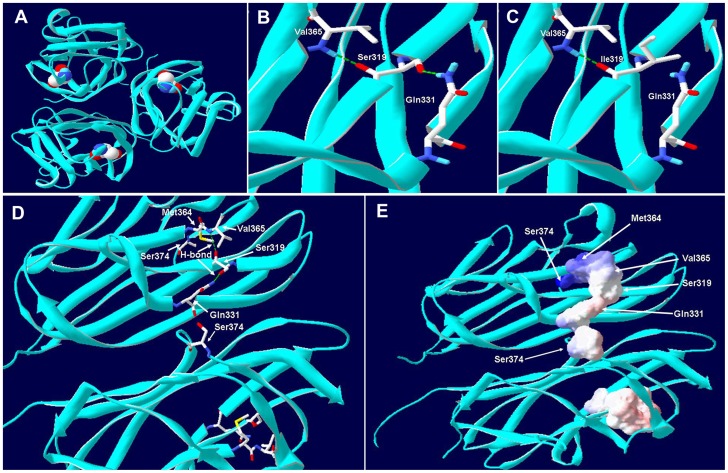
*In silico* analysis of p.Ser319Ile mutation on protein structure. (A) Model showing homo trimer of TNF homology domain of wild type EDA1 protein Ser319 residue is indicated by space filling structure. (B) Ser319 form hydrogen bond (*green line*) with Gln331 and Val365 (C) After getting substituted Ile319 unable form hydrogen bond (*green line*) with Gln331 (D) Location of five amino acid (Ser319, Gln331, Met364, Val365 and Ser374) with in the monomer-monomer interface of two EDA1 monomer, mutations in those amino acids are associated with non-syndromic tooth agenesis. (C) Surface electrostatic potentiality of those five residues shows an overlapping region that may involved in receptor binding activity. All the images are produced through Swiss-PdbViewer 4.1.0 software using PDB file 1RJ7.

Localization of Ser319 on previously reported three dimensional structure of EDA TNF homology region (PDB ID: 1RJ7) showed that this amino acid is located towards the periphery of EDA monomer with in the monomer-monomer interface (Figure 5A). Stick model and surface electrostatic potential calculation through *in silico* analysis showed that four amino acid residues (Gln331, Met364, Val365 and Ser374) for which mutation have been known to cause non-syndromic congenital tooth agenesis along with Ser319 resides in close proximity to each other and form overlapping surface with slightly negative potential (Figure 5D, E). Out of those five residues Ser319 form hydrogen bonding with Gln331 and Val365. So due to the novel Ser319Ile mutation the mutant Ile319 unable to form hydrogen bond with Gln331 (Figure 5B, C).

## Discussion

In our present study we have identified a novel c.956G>T mutation in *EDA1* gene in an Indian family with X-linked dominant type of non-syndromic tooth agenesis affecting both primary and permanent dentition. While tooth agenesis was mainly restricted to incisors and canines in all the affected females, affected male child also showed agenesis of deciduous molar and permanent premolar which indicates that the male child is more susceptible to the pathogenic affect of the mutation. Careful clinical study unable to identify any features of Ectodermal dysplasia or mental retardation, as mentioned in available literatures in any of the affected family members including proband [27]. Initially the pattern of inheritance was seems to be autosomal dominant type but sequence of all the coding exons and exon-intron boundaries of two major candidate genes *viz. PAX9* and *MSX1* failed to reveal any pathogenic mutation, leading to possible association of novel candidate gene/s with the disease phenotype. Whole genome sequence analysis by next generation or third generation sequencing techniques has become a powerful tool for identification of novel gene or mutation underlying disease condition.

Two of the affected family members (III2 and IV4) selected for whole genome sequencing showed 119 novel non-synonymous DNA sequence variants compared to the unaffected and those were distributed in 117 different genes across the genome. However, among those 119 sequence variants a c.956G>T mutation in X-linked *EDA1* was the unique one among six candidate genes (*viz.PAX9, MSX1, AXIN2, WNT10A, EDAR* and *EDA1*) associated with non-syndromic tooth agenesis known so far.

Functional EDA1 is a type-II trimeric transmembrane protein belonging to TNF ligand superfamily. It has four distinct functional parts; an N-terminal intracellular domain, a collagen like repeat domain, a furin cleavage site and a C-terminal TNF homology domain [28]. The C-terminal domain subsequently cleaved off at the furin cleavage site and binds with EDAR and subsequently regulates cellular physiology by NF-κB pathway [29]. The amino acid variation (p.S319I) observed in DEN12 family is located with in the TNF homology domain which shows 100% amino acid sequence similarity with mouse Eda. Several studies recognize TNF homology domain along with collagen sub-domain as a mutational hotspot for EDA protein. Sequence homology modeling of EDA protein using PDB entries showed that TNF homology domain has two distinct regions, one consists of seven trimer forming residues (H252, F302, Y304, Y347, C352, F379 and I383) and another consists of five receptor binding residues (S275, I277, S319, 326E and 328P). So far 74 mutations have been identified out of which 47 are located in conserved TNF homology domain. 13 mutations among those 47 are responsible for non-syndromic tooth agenesis.[30], [31], [32], [33] Out of those, four residues (Gln331, Met364, Val365 and Ser374) and Ser319 (present study) can form a cluster with slightly negative surface potential. This being located with in the monomer-monomer interface plays critical role during receptor binding activity of EDA and several other TNF domain containing protein.[34], [25], [35] Several studies showed that mutations which affect the structural stability of EDA protein mainly contribute in X linked hypohidrotic ectodermal dysplasia (XLHED) on the other hand mutations which slightly alter the receptor binding activity have their role in milder form of XLHED and/or non-syndromic tooth agenesis. Ser319, which is a part of five receptor binding resides as observed from NCBI Conserve Domain Database (CDD), also form hydrogen bond with Gln331 and Val365 (mutation in this two residues are reported to be associated with non-syndromic tooth agenesis). Due to mutation in Ser to Ile, hydrogen bond between Ser319 and Gln331 get disrupted and the receptor binding residues thus reduced from five to four.

In this family a total of three females and one male showed congenital tooth agenesis and all of them have c.956G>T mutation in X linked *EDA* gene resulting p.Ser319Ile change in EDA1 protein. Unlike other X linked phenotype, in which most of the females remain protected from the effect of X-linked mutation due to X inactivation phenomenon, in our study all the females carrying this mutation (c.956G>T) in heterozygous condition (X^G^X^T^) showed mutant phenotype along with the male child who has only one copy of X linked *EDA* and so showed the mutant type (X^T^Y). Although this mutation shows complete penetrance but the expressivity differs not only between male and female, which is obvious due to presence of mutant genotype in hemizygous (X^T^Y) condition, but also among females. To explain this rear behavior of this X-linked mutation careful study of available literatures had been carried out. In one study, a Chinese family with p.Asp316Gly mutation in EDA1 protein was found to be associated with non-syndromic congenital tooth agenesis affecting all tooth type. All the children, three females and one male showed hypodontia phenotype with differential expressivity.[36] This affection status closely resembles to that of the family we had identified. Further, functional analysis of p.Asp316Gly along with five other mutations associated with non-syndromic tooth agenesis showed that this mutation, unlike other five, drastically reduces interaction between EDA receptor (EDAR) and mutant EDA1 protein.[31] These two studies clearly indicate that mutation(s) which severely affect the receptor binding activity of EDA protein may show almost complete penetrance for male and female in spite of the X inactivation. One possible explanation of this may lies during the course of tooth development. In female embryo there is equal chance of inactivation either of mutant or of wild type gene (eg. *EDA*) located in X chromosome leading to mosaicism. Thus at the site of tooth development amount of bioactive protein will become almost half if the activity of the mutant protein reduced down to near zero which subsequently leads to milder form of hypodontia phenotype in females compared to males with only the mutant copy of the gene.

Thus in our study the novel p.Ser319Ile mutation located in a putative receptor binding region may almost completely abolish the receptor binding activity of mutant EDA1 protein. In females (DEN12 III2, IV1 and IV2) due to mosaic pattern of X-inactivation the pathogenic activity of this mutation could be diluted leading to milder phenotype compared to the affected male (DEN12 IV4), with single X chromosome bearing the mutant *EDA.* In a resent study by Piccione *et al.* (2012), identify mutation in same amino acid position associated with X-linked hypohidrotic ectodermal dysplasia with keratoconus.[37] Further expression and functional analysis of p.Ser319Ile mutation could provide direct evidence regarding the pathogenic property of this mutation.

### Accession Numbers

c.956G>T mutation in human gene EDA transcript variant 1: NCBI ClinVar database (SCV000148371) and NCBI dbSNP database (rs483352804).

### Web Resources

The URLs for data presented herein are as follows:

NCBI BLAST tool, http://blast.ncbi.nlm.nih.gov/Blast.cgi.

UCSC-BLAT tool, http://genome.ucsc.edu/cgi-bin/hgBlat.

Polymorphism phenotype-2, http://genetics.bwh.harvard.edu/pph2/.

NCBI Conserved domain search tool, http://www.ncbi.nlm.nih.gov/Structure/cdd/wrpsb.cgi.

Online Mendelian Inheritance in Man (OMIM), http://www.ncbi.nlm.gov/Omim/.

ClustalW2 software, http://www.ebi.ac.uk/Tools/msa/clustalw2/.

Primer3web software, http://primer3.ut.ee/.

## Supporting Information

Table S1 **Novel non-synonymous nucleotide variations detected in two affected individuals (DEN12-III2, IV4) through Whole Genome Sequencing.**
(XLS)
